# The Role of Telemedicine Centers and Digital Health Applications in Home Care: Challenges and Opportunities for Family Caregivers

**DOI:** 10.3390/healthcare14010136

**Published:** 2026-01-05

**Authors:** Kevin-Justin Schwedler, Jan Ehlers, Thomas Ostermann, Gregor Hohenberg

**Affiliations:** 1 Stabsstelle für Digitalisierung und Wissensmanagement, Hochschule Hamm-Lippstadt, 59063 Hamm, Germany; gregor.hohenberg@hshl.de; 2 Fakultät für Gesundheit, Universität Witten-Herdecke, 58455 Witten, Germany; jan.ehlers@uni-wh.de (J.E.); thomas.ostermann@uni-wh.de (T.O.)

**Keywords:** telemedicine, family care, digital health applications, health monitoring, home care

## Abstract

Background/Objectives: Home care plays a crucial role in contemporary healthcare systems, particularly in the long-term care of people with chronic and progressive illnesses. Family caregivers often experience substantial physical, emotional, and organizational burden. Telemedicine and digital health applications have the potential to support home care by improving health monitoring, communication, and care coordination. However, their use among family caregivers remains inconsistent, and little is known about how organizational support structures such as telemedicine centers influence acceptance and everyday use. This study aims to examine the benefits of telemedicine in home care and to evaluate the role of telemedicine centers as supportive infrastructures for family caregivers. Methods: A mixed-methods design was applied. Quantitative data were collected through an online survey of 58 family caregivers to assess the use of telemedicine and digital health applications, perceived benefits, barriers, and support needs. This was complemented by an in-depth qualitative case study exploring everyday caregiving experiences with telemedicine technologies and telemedicine center support. A systematic literature review informed the theoretical framework and the development of the empirical instruments. Results: Most respondents reported not using telemedicine or digital health applications in home care. Among users, telemedicine was associated with perceived improvements in quality of care, particularly through enhanced health monitoring, improved communication with healthcare professionals, and increased feelings of safety and control. Key barriers to adoption included technical complexity, data protection concerns, and limited digital literacy. Both quantitative findings and the qualitative case study highlighted the importance of structured support. Telemedicine centers were perceived as highly beneficial, providing technical assistance, training, coordination, and ongoing guidance that facilitated technology acceptance and sustained use. Conclusions: Telemedicine and digital health applications can meaningfully support home care and reduce caregiver burden when they are embedded in supportive socio-technical structures. Telemedicine centers can function as central points of contact that enhance usability, trust, and continuity of care. The findings suggest that successful implementation of telemedicine in home care requires not only technological solutions but also accessible organizational support and targeted training for family caregivers.

## 1. Introduction

Home care plays a crucial role in contemporary healthcare systems, particularly in the long-term care of people with chronic and progressive illnesses. Family caregivers assume extensive responsibilities that are frequently associated with substantial physical, emotional, and organizational burden [1,2,3]. Numerous studies have shown that prolonged caregiving is linked to increased stress, reduced quality of life, and adverse mental health outcomes, especially in dementia care, palliative care, and multimorbidity contexts [1,2,4,5]. Demographic change and population aging further intensify these challenges, underlining the growing need for effective and sustainable support structures in home care settings.

Caregiver burden and resource-oriented perspectives

From a theoretical perspective, caregiver burden is commonly conceptualized as the result of an imbalance between care-related demands and available personal, social, and structural resources. Resource-oriented models assume that additional informational, emotional, and organizational resources can buffer caregiving strain and improve caregivers’ coping capacity. Digital health technologies, including telemedicine and digital health applications, have increasingly been discussed as such potential resources, as they may enhance access to professional support, facilitate communication, and improve care coordination [4,6,7].

Systematic reviews and meta-analyses indicate that telehealth interventions can reduce caregiver burden and improve mental health outcomes, particularly among caregivers of people with dementia, chronic diseases, and those receiving palliative care [1,2,3,5]. However, the reported effects vary considerably across studies, suggesting that technological solutions alone are insufficient and that contextual and organizational factors play a crucial role in determining their effectiveness [8,9].

Telemedicine and digital health applications in home care

Telemedicine refers to the provision of healthcare services across geographical distances through information and communication technologies, enabling remote monitoring, consultation, and professional support [10]. Digital health applications encompass a broader category of software-based tools, including mobile health applications, telemonitoring systems, and certified digital health applications such as German Digitale Gesundheitsanwendungen (DiGA), which support health monitoring, self-management, and communication with healthcare providers [8,11]. In home care contexts, these concepts overlap substantially, as digital health applications are often embedded within telemedicine services or used alongside them.

Empirical studies suggest that telemedicine and digital health applications can improve symptom monitoring, enhance communication with healthcare professionals, and increase perceived safety among family caregivers, particularly in rural or underserved regions [6,7,12,13]. Nevertheless, adoption and sustained use remain inconsistent, indicating the need for a better understanding of the conditions under which these technologies are effectively integrated into everyday care routines.

Technology acceptance models (TAM and UTAUT)

To explain the adoption and use of telemedicine technologies, established technology acceptance models provide an important theoretical lens. The Technology Acceptance Model (TAM) and the Unified Theory of Acceptance and Use of Technology (UTAUT) posit that technology use is primarily determined by perceived usefulness, perceived ease of use, social influence, and facilitating conditions [14]. Applied to family caregiving, these models suggest that acceptance of telemedicine depends not only on technical functionality but also on the availability of guidance, training, and ongoing professional support.

Previous studies have identified technical complexity, limited digital literacy, data protection concerns, and insufficient professional assistance as major barriers to telemedicine adoption among family caregivers [15,16,17]. These findings highlight the importance of facilitating conditions and structured support as central determinants of technology acceptance and sustained use in home care contexts.

Socio-technical support structures and telemedicine centers

Beyond individual acceptance, recent research emphasizes socio-technical perspectives that conceptualize digital health technologies as embedded within broader organizational and institutional contexts. From this perspective, successful implementation depends on the interaction between users, technologies, and supportive infrastructures [18,19]. Telemedicine centers can be conceptualized as such socio-technical support structures, functioning as centralized organizational units that connect family caregivers, healthcare professionals, and digital technologies.

Telemedicine centers typically provide technical support, training and digital literacy assistance, care coordination, and continuous monitoring and feedback services [20,21]. Evidence from program evaluations and qualitative studies suggests that such centers may increase trust in digital solutions, improve continuity of care, and enhance caregivers’ confidence in using telemedicine technologies [12,21,22]. However, empirical evidence on how family caregivers experience the role of telemedicine centers in everyday home care remains limited.

Study aim and research questions

The aim of this paper is to examine the benefits of telemedicine in home care and to evaluate the role of telemedicine centers as supportive structures that can make the use of telemedicine systems safer, more reliable, and more accessible for family caregivers [12]. Specifically, the study investigates the extent to which telemedicine and digital health applications are used by family caregivers, the perceived benefits of these technologies, and the potential contribution of telemedicine centers to optimizing their use.

The central research questions are therefore as follows: Are telemedicine and digital health applications used by family caregivers, and if so, what specific benefits do they offer? Can a telemedicine center further improve the use and acceptance of these technologies? By combining quantitative survey data with an in-depth qualitative case study, this mixed-methods study aims to provide a comprehensive understanding of current practices, challenges, and support needs related to telemedicine in home care.

## 2. Materials and Methods

### 2.1. Study Design

The study follows a mixed-methods design combining quantitative and qualitative approaches to address the research questions comprehensively. This design was chosen to capture both the breadth of telemedicine use among family caregivers and the depth of individual experiences related to organizational support structures.

The research design consists of two complementary components:

1. A quantitative online survey to assess usage patterns, perceived benefits, barriers, and support needs related to telemedicine and digital health applications among family caregivers; 

2. A qualitative case study to explore in depth how telemedicine technologies and telemedicine center support are experienced and integrated into everyday home care.

The mixed-methods approach enables triangulation between empirical findings and existing evidence. The systematic literature review provided the conceptual and theoretical foundation for both components and guided the development of the questionnaire and the qualitative interview guide. The integration of quantitative and qualitative findings allows for a nuanced interpretation of telemedicine use in home care and the role of telemedicine centers as socio-technical support structures.

The study was explicitly designed as an exploratory investigation using mixed methods to identify usage patterns, perceived benefits, and support needs related to telemedicine in home care, rather than to test predefined causal hypotheses.

### 2.2. Literature Review

The theoretical and empirical foundation of the study was established through a systematic literature review conducted in accordance with established guidelines for transparent reporting. The databases PubMed, CINAHL, Web of Science, and Google Scholar were searched between January and March 2025.

The search strategy combined terms related to telemedicine and digital health with terms referring to family caregiving and home care contexts:

(“telemedicine” OR “telehealth” OR “digital health application” OR “DiGA”) AND

(“family caregivers” OR “informal caregivers”) AND

(“home care” OR “long-term care” OR “telemedicine center”)

In this study, digital health applications are defined as software-based health interventions used to support health monitoring, self-management, communication, or care coordination in home care settings. This includes mobile health applications, telemonitoring systems, and certified German Digitale Gesundheitsanwendungen (DiGA). Telehealth refers, more specifically, to the remote delivery of healthcare services. Both concepts were included in the search strategy because they substantially overlap in home care contexts and are often jointly implemented in caregiving practice.

The selected search terms were chosen to balance sensitivity and specificity. While additional terms such as “eHealth”, “mHealth”, or “remote patient monitoring” are frequently used in the literature, these concepts largely overlap with telemedicine and telehealth in home care research. Restricting the search to a limited number of umbrella terms reduced the risk of retrieving predominantly technical studies with limited relevance to family caregivers, while ensuring comprehensive coverage of relevant interventions.

**Figure 1:**
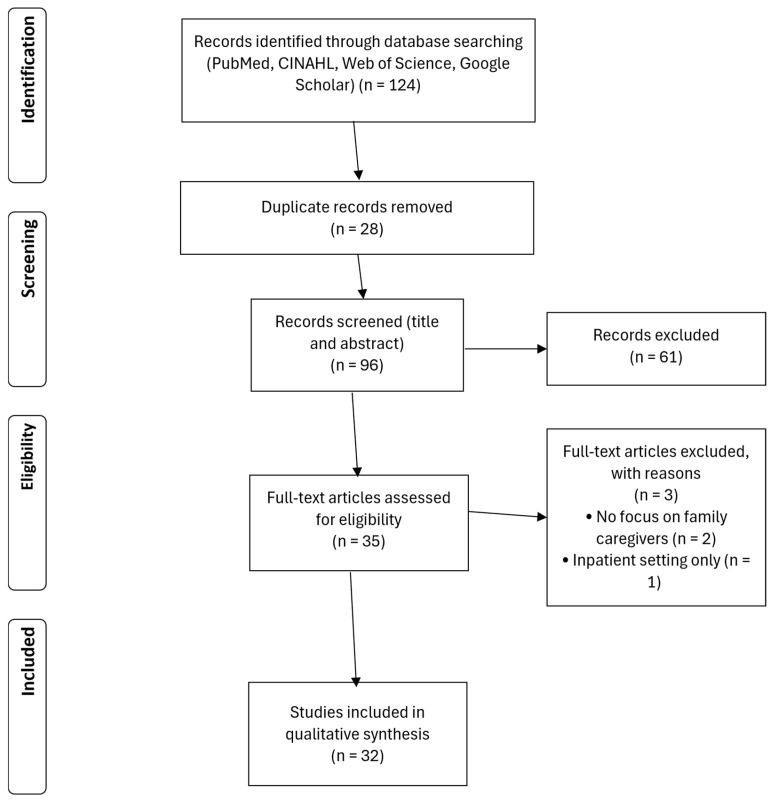
PRISMA-Flowchart.

The initial database search yielded 124 records. After removal of 28 duplicates, 96 titles and abstracts were screened for relevance. At this stage, 61 records were excluded because they did not focus on family caregivers, addressed exclusively inpatient settings, or had a purely technical focus. The remaining 35 full-text articles were assessed for eligibility. Following full-text screening, 3 articles were excluded due to insufficient relevance to home care or the absence of a caregiver perspective. In total, 32 studies met all inclusion criteria and were included in the final synthesis. The study selection process is illustrated in a PRISMA flow diagram (Figure 1).

The purpose of the systematic literature review was not to constitute an independent review study but to provide a structured theoretical and empirical foundation for the mixed-methods design. The review identified key concepts, usage patterns, perceived benefits, barriers, and organizational support structures related to telemedicine use by family caregivers. These findings directly informed the development of the quantitative questionnaire and the guiding questions of the qualitative case study, thereby synchronizing the literature review with the empirical components of the study.

The analysis revealed three central thematic areas:

1. Use and acceptance of telemedicine technologies

Studies indicate that family caregivers accept telemedicine solutions primarily when they are perceived as suitable for everyday use, easy to operate, and reliable [15,16,20]. Major barriers include technical complexity, data protection concerns, and insufficient training [14,15].

2. Effects of telemedicine support

Several studies demonstrate reductions in caregiver burden and stress, improved communication with healthcare professionals, and increased subjective feelings of safety among family caregivers [1,7,17,21,23].

3. Role of telemedicine centers

Recent research highlights the importance of centralized telemedicine contact points that support family caregivers with implementation, technical use, and problem-solving [17,20,21,22,23]. Such structures can significantly improve continuity of care, coordination with physicians, and digital health literacy among family members [18].

These findings formed the theoretical basis for the development of the questionnaire and the guiding questions of the qualitative case study.

### 2.3. Quantitative Online Survey

#### 2.3.1. Development of the Questionnaire

The questionnaire was developed to record the following:

- The use and evaluation of telemedicine technologies;
- The perceived support provided by telemedicine centers;
- The needs and expectations of family caregivers.

The items were based on technology acceptance models (TAM, UTAUT2) and empirical preliminary work on DiGAs in nursing care [5,6].

The questionnaire comprised 32 items, divided into six subject areas (see Table 1).

#### 2.3.2. Validation and Pretest

The content was validated by a panel of three experts (nursing science, telemedicine, family counseling). The calculated content validity index (S-CVI) was 0.91, indicating high content validity.

A pretest with five caregiving relatives was used to test comprehensibility. Feedback led to minor linguistic adjustments and a clearer distinction between “digital application” and “telemedicine technology.” Given the exploratory nature of the study and the limited sample size, the psychometric evaluation focused on content validity and internal consistency, while no factor analysis procedures were performed.

#### 2.3.3. Implementation

The online survey was conducted with 58 family caregivers. Recruitment took place via caregiving courses. Conditions of participation are as follows:

- Active home care of a family member with a chronic illness;
- Minimum age of 18;
- Consent to anonymous data collection.

Participation was voluntary and anonymous.

The achieved sample size of 58 family caregivers was considered appropriate for the exploratory aim of the quantitative component. The study was designed to identify usage patterns, perceptions, and support needs related to telemedicine and telemedicine centers in home care, rather than to test predefined hypotheses.

Given the limited accessibility of family caregivers as a target population and the heterogeneity of caregiving situations, a pragmatic recruitment approach was applied. Comparable exploratory studies in the field of digital health and informal caregiving have used similar sample sizes to generate initial empirical evidence and to inform the development of future, larger-scale studies. Recruitment via caregiving courses may have favored family caregivers who are more motivated, more health literate, or more open to digital technologies, which may have led to selection bias.

#### 2.3.4. Data Analysis

The quantitative data were analyzed using SPSS Statistics Version 29 (IBM Corp., Armonk, NY, USA).

- Descriptive analyses: frequencies, means, standard deviations.
- Reliability test: Cronbach’s α = 0.87.
- Correlations between variables (e.g., use of technology and perceived support from a TMZ) were examined using χ^2^ tests and *t*-tests.
- Open-ended responses were categorized qualitatively according to Mayring.

To control for confounding factors such as age, education, and care intensity, multivariable models such as logistic and linear regressions were used. These models make it possible to examine the independent effects of various variables on the use and acceptance of telemedicine technologies. For example, logistic regression shows that higher education (OR = 2.45, 95% CI: 1.30–4.60) and lower care intensity (OR = 0.75, 95% CI: 0.60–0.95) are significantly associated with a higher probability of using telemedicine technologies (*p* < 0.05). These findings underscore the importance of demographic and contextual factors in the implementation and use of digital health solutions.

Key aspects of the analysis:

- Proportion of family caregivers using telemedicine.
- Perceived relief provided by technologies.
- Potential and need for a telemedicine center.

### 2.4. Qualitative Case Study

#### 2.4.1. Objective and Selection

The qualitative case study aimed to examine in depth the practical benefits and challenges associated with the use of a telemedicine center in home care. The focus was on understanding everyday caregiving processes, perceived support structures, and experienced changes resulting from telemedicine use.

A single, information-rich case was deliberately selected to allow an in-depth exploration of mechanisms, meanings, and contextual factors related to telemedicine center support. The participating family caregiver was chosen because of their continuous use of a telemedicine monitoring system and active engagement with a telemedicine center in the care of a person with dementia. This approach is consistent with qualitative research principles, where analytical depth and contextual understanding are prioritized over sample size and statistical generalizability. The case study was not intended to provide representative evidence, but rather to illustrate mechanisms, processes, and contextual factors that contribute to the interpretation of the quantitative results.

#### 2.4.2. Data Collection

Data was collected in June 2025 using a semi-structured guided interview based on the following key questions:

1. Daily care routine before using technology;
2. Daily care routine after using technology;
3. Changes brought about by technology;
4. Role and support provided by the telemedicine center;
5. Impact on sense of security;
6. Comparison: with vs. without telemedicine center;
7. Specific situations of improvement.

The interview was recorded, transcribed, and pseudonymized.

#### 2.4.3. Analysis

The qualitative evaluation was carried out using structured content analysis according to Mayring (2015).

Categories were formed inductively, relating to the effect of telemedicine technologies and the importance of the telemedicine center.

Particular attention was paid to the following:

- Relief in everyday nursing care;
- Improvement in communication with specialist staff;
- Technical and emotional support from the TMZ.

### 2.5. Ethical Aspects

The study received a positive ethics vote from the University of Witten/Herdecke (vote no. 2025-03-27).

All participants were informed in writing about the aim, procedure, and data protection and gave their consent.

Data was stored in accordance with the GDPR, pseudonymized, and used exclusively for research purposes.

### 2.6. Limitations

- Small sample size (n = 58) limits generalizability.
- Recruitment via training courses may lead to a technology-biased sample.
- The case study represents a single perspective.
- Reliability tests are based on an initial round of surveys.

The use of telemedicine and digital health applications, as well as the perceived benefits, were assessed based on self-reported information, which may be subject to bias due to memory gaps and social desirability effects. The recruitment strategy may have led to an overrepresentation of digitally interested caregivers, meaning that the prevalence and acceptance of telemedicine observed in this study may be higher than in the general population of family caregivers. Future studies with larger samples should examine construct validity using exploratory or confirmatory factor analyses to further improve measurement quality. Despite these limitations, the results provide valuable insights into the practical role of telemedicine centers as a key structure for supporting family caregivers in the digital care context.

## 3. Results

### 3.1. Sample Characteristics and Use of Digital Technologies

**Figure 2:**
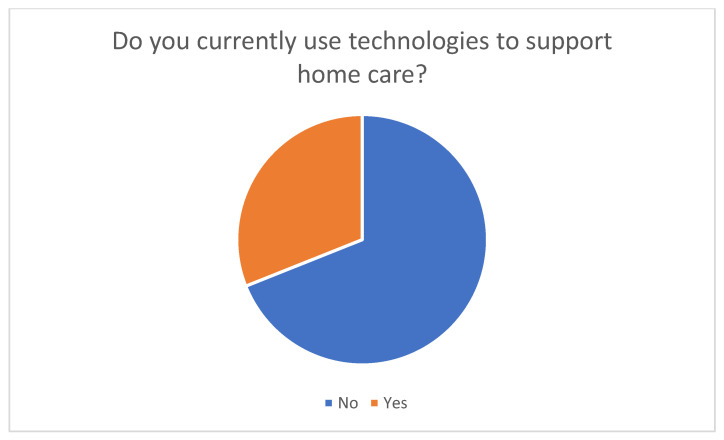
Current use of technical support services in home care.

The online survey included 58 family caregivers. The results show that the majority of respondents do not currently use digital or telemedicine technologies to support home care. Forty participants (69%) reported not using any technologies, while 18 participants (31%) stated that they use telemedicine or digital health applications in everyday care (Figure 2). This indicates that the use of digital and telemedical solutions among family caregivers is still limited.

**Figure 3:**
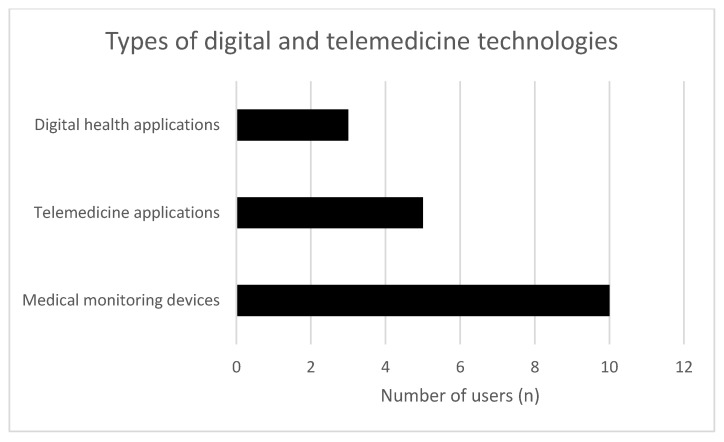
Types of digital and telemedicine technologies.

Among those who reported using technologies, different types of applications and devices were used. The most frequently mentioned were medical monitoring devices, such as blood pressure or blood glucose monitors, which were used by 10 of the 18 users. Telemedicine applications, including video consultations and remote medical contact, were reported by 5 users. Digital health applications, for example, for medication management or appointment scheduling, were used by 3 respondents (Figure 3).

**Figure 4:**
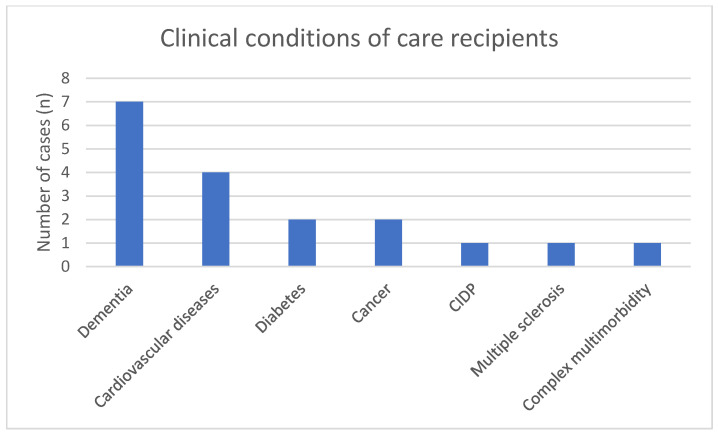
Clinical conditions of care recipients.

The persons receiving care were affected by a wide range of clinical conditions. Dementia was the most frequently reported condition (n = 7), followed by cardiovascular diseases (n = 4). Other conditions included diabetes (n = 2), cancer (n = 2), chronic inflammatory demyelinating polyneuropathy (CIDP) (n = 1), multiple sclerosis (MS) (n = 1), and complex combinations of dementia, Parkinson’s disease, diabetes, and other conditions (n = 1) (Figure 4). These findings illustrate the heterogeneity of care situations in which digital and telemedicine technologies are used.

### 3.2. Patterns of Use and Selection Criteria

**Figure 5:**
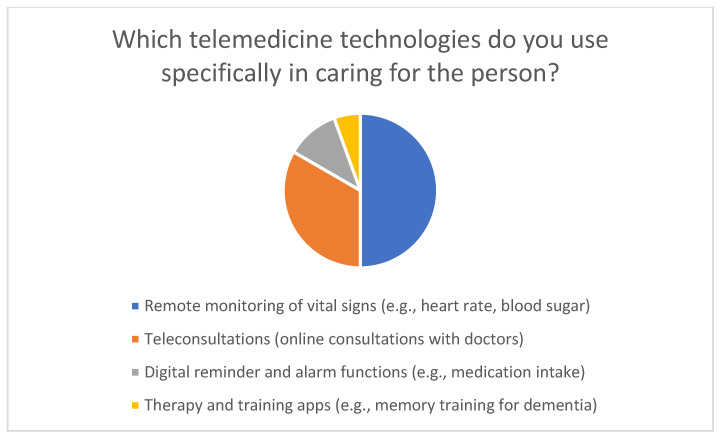
Telemedicine technologies used in home care.

Family members who take on caregiving tasks reported using telemedicine technologies in various ways to support daily care (Figure 5). The most common use was remote monitoring of vital signs such as heart rate or blood sugar levels, which was used by about half of the technology users. About one-third of respondents reported using teleconsultations with doctors. Digital reminder and alarm functions, such as for taking medication, as well as therapy and training apps, such as memory training applications for people with dementia, were used by about one-fifth of respondents.

Respondents named a variety of specific DiGAs and software solutions, including digital monitoring systems, video applications for communication with healthcare professionals, memory training apps, and applications for documenting and tracking symptoms. The main functions of these applications were monitoring vital signs, facilitating communication with physicians, and supporting the organization and management of care (Table 2).

The selection of specific applications was influenced by several factors. Family caregivers reported choosing technologies that facilitate communication and treatment, particularly when video functions or digital monitoring systems enabled direct interaction with the medical team. Other important selection criteria were the support and satisfaction of the person receiving care, speed and simplicity of use, and the availability of the technologies. Security and having a clear overview of health data also played an important role. In addition, some applications were selected because they offered motivating elements, such as memory games or exercise-promoting functions (Table 2). Monitoring technologies are used more frequently for cardiovascular and metabolic diseases, while memory and cognitive training applications were mainly used in dementia care, which indicates the disease-specific suitability of various digital tools.

### 3.3. Integration into Daily Care and Challenges

The integration of DiGAs and software solutions into everyday care was perceived differently by respondents. Several family caregivers reported an initial adjustment period, during which they needed time to become familiar with the technologies. This was particularly relevant for older caregivers or those with limited digital experience (Table 3).

Data protection concerns were mentioned by some respondents as a barrier that initially made the use of digital technologies more difficult. Technical handling was described as unproblematic when applications were user-friendly and self-explanatory. In addition, some caregivers reported that a positive professional attitude toward caregiving and new technologies facilitated integration into daily routines (Table 3).

The systematic quantification and classification of the obstacles revealed that data protection concerns and technical complexity were the most common barriers. A severity rating on a scale of 1 (low) to 5 (high) showed that data protection concerns had an average severity rating of 4.2 and technical complexity had a severity rating of 3.8. These results highlight the need for targeted interventions and training to overcome these barriers and facilitate the integration of DiGAs and software solutions into everyday care.

**Figure 6:**
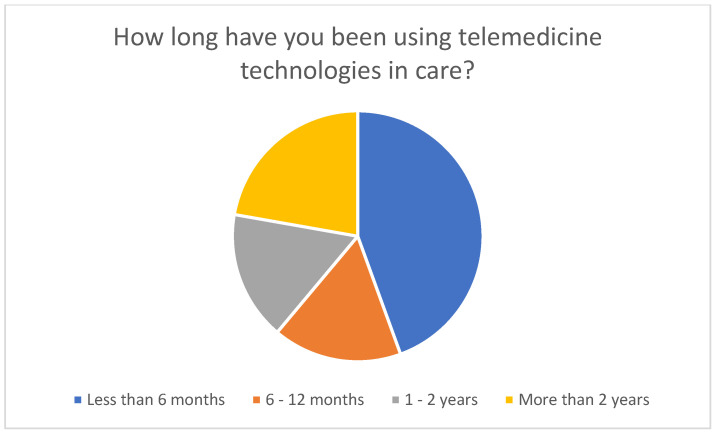
Duration of use of telemedicine technologies.

The duration of technology use varied among respondents (Figure 6). Eight users had been using telemedicine technologies for less than six months, three for six to twelve months, three for one to two years, and four for more than two years. This distribution indicates that a considerable proportion of users had only recently started using digital technologies, suggesting a gradual increase in acceptance and dissemination.

### 3.4. Perceived Effects on Quality of Care and Communication

**Figure 7:**
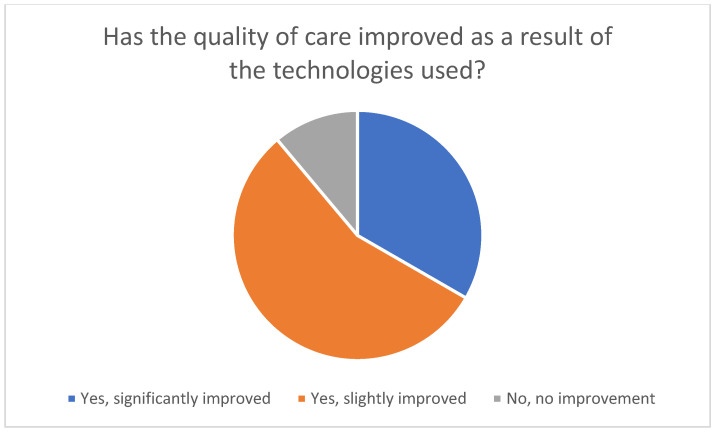
Perceived change in the quality of care through the use of telemedicine technologies.

Most family caregivers who used telemedicine technologies perceived an improvement in the quality of care (Figure 7). Sixteen of the 18 users reported a positive effect, with ten describing a slight improvement and six reporting a significant improvement. Two respondents stated that they had not noticed any improvement.

The most frequently reported positive effect was improved health monitoring, which was mentioned by 11 users. Respondents emphasized that continuous and accurate monitoring of vital signs enabled better control of health status and early detection of changes. Greater independence of the person receiving care was reported by three users, as technologies supported the performance of everyday tasks. A reduction in stress and uncertainty for family caregivers was also mentioned by three respondents. Improved communication with healthcare providers was reported by one user.

Respondents provided specific examples of significant improvements, including the immediate availability of updated vital signs for the medical team, early detection of falls, reminders for medication intake and medical appointments, continuous monitoring after medical procedures, motivational effects of memory training games, and improved treatment adjustment based on documented symptoms and health data (Table 4).

The use of telemedicine technologies also affected communication with physicians and nursing staff. Respondents reported easier and more efficient communication due to the direct transmission of health data. Time savings were noted, as fewer explanations and follow-up questions were required. Some respondents stated that physicians were better prepared for consultations because relevant data were available in advance. Comprehensive documentation and an improved overview of health information were also described as beneficial for communication and care coordination (Table 4).

### 3.5. Evaluation, Acceptance, and Recommendations

**Figure 8:**
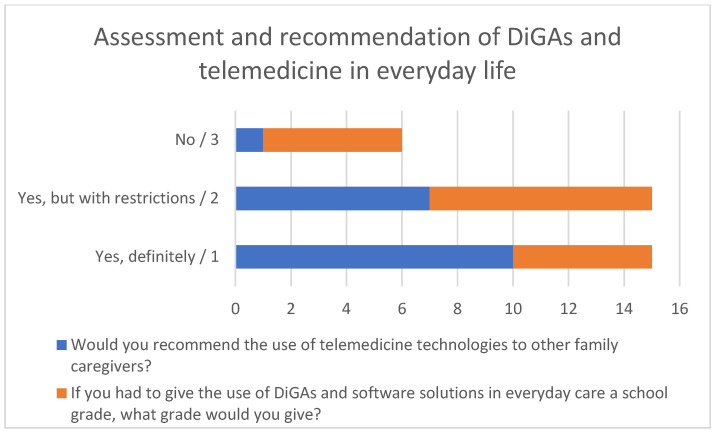
Assessment and recommendation of DiGAs and telemedicine in everyday life.

Most family caregivers reported positive overall experiences with telemedicine technologies (Figure 8). Ten of the 18 users stated that they would definitely recommend the use of these technologies, while seven would recommend them with reservations. One respondent indicated that they would not recommend their use.

When asked to evaluate DiGAs and software solutions in everyday care using a school grading system, five respondents assigned the highest grade, eight gave a grade of two, and five gave a grade of three. Overall, the technologies were rated as good to very good.

Additional comments highlighted that telemedicine technologies were perceived as helpful and convenient, particularly for keeping track of health information and providing a sense of security. Some respondents noted that the technologies required an initial familiarization phase but worked well after this period. Motivational aspects, such as encouraging physical activity or cognitive training, as well as practical support for appointment and medication planning, were also emphasized (Table 5).

### 3.6. Expectations Regarding Telemedicine Centers

**Figure 9:**
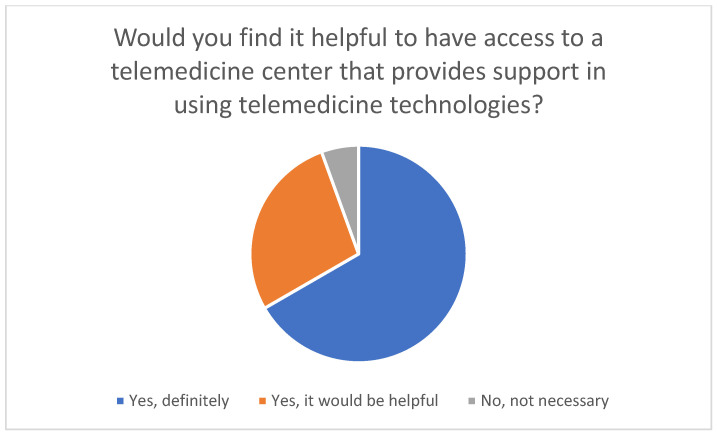
Perceived usefulness of a telemedicine center in supporting the application of telemedicine technologies.

The majority of family caregivers reported that access to a telemedicine center would be helpful (Figure 9). Twelve of the 18 users stated that such a center would definitely be helpful, while five indicated that it would be helpful. Only one respondent felt that a telemedicine center was not necessary.

Respondents expressed a clear need for technical support, including assistance with setting up devices and software and rapid help in case of technical problems. Training and education on how to use technologies effectively and advice on selecting appropriate applications for specific conditions were also frequently mentioned. In addition, respondents wished for brief explanations, tips, and support in setting reminders and managing calendars, particularly for older users (Table 6).

**Figure 10:**
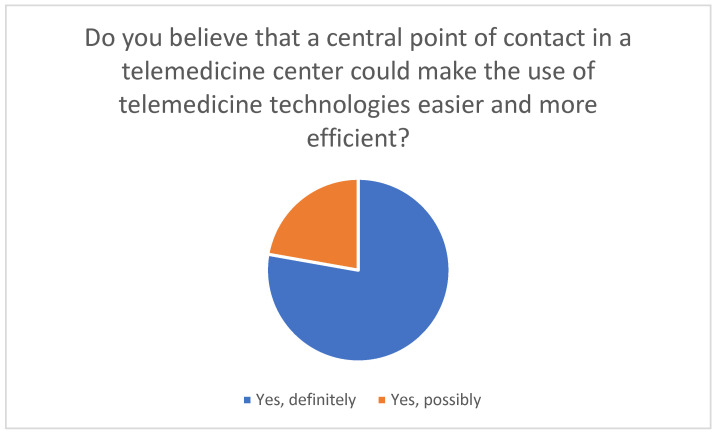
Perceived benefits of a central point of contact at the telemedicine center.

A central point of contact was perceived as advantageous, as it could provide direct help and advice, increase security and trust, and improve efficiency and motivation in using technologies (Figure 10). Further perceived benefits included transparent communication with healthcare providers, fast and uncomplicated support, and continuous consultation to improve the quality of care.

Concerns regarding a telemedicine center mainly related to potential costs, data protection, and the technical complexity of digital solutions, especially for older people. Despite these reservations, most respondents believed that regular consultation with a telemedicine center could help overcome difficulties and contribute to better communication, error management, and confidence in using telemedicine technologies (Table 7).

The majority of respondents expected that regular consultations with a telemedicine center would further improve the quality of care, particularly through better communication, improved error management, and a clearer overview of health data and trends. Regular feedback was seen as helpful for maintaining motivation, improving documentation, and responding more quickly to problems that arise. To achieve these benefits, participants emphasized that a telemedicine center should be easily accessible, centrally organized, and user-friendly, providing clear information, simple step-by-step instructions, and rapid support across multiple communication channels (Table 8).

### 3.7. Qualitative Case Study

To complement the quantitative results, a qualitative case study was conducted to provide deeper insight into the practical use of telemedicine technologies and the support provided by a telemedicine center. The case study is based on a guided interview with a family caregiver who has been caring for her husband with dementia for several years.

Before the use of telemedicine technologies, daily care was described as highly stressful. Regular occupational therapy sessions outside the familiar home environment caused anxiety for the care recipient and placed a considerable burden on both partners. Since the introduction of telemedicine technologies, cognitive training can be carried out in familiar surroundings, which has led to a noticeable reduction in stress and improved concentration and training outcomes.

The telemedicine center played a central role by providing detailed instruction, continuous availability, and rapid assistance in case of technical problems or uncertainties. This support increased the caregiver’s sense of security and confidence in using the technologies. According to the caregiver, comparable results would not have been achieved without the support of the telemedicine center.

Overall, the case study illustrates how the combination of telemedicine technologies and structured support can reduce stress and improve the quality of home care, thereby reinforcing the findings of the quantitative survey.

## 4. Discussion

### 4.1. Interpretation of the Main Findings

The results of the present study indicate that telemedicine and digital health applications have considerable potential to support home care and to reduce the burden on family caregivers [1,2,4,6,7]. Although the overall use of such technologies among respondents was still limited, family caregivers who did use telemedicine reported predominantly positive effects, which is consistent with previous systematic reviews and meta-analyses in different caregiving contexts [1,2,3,7]. The results of the study were clearly attributed to specific constructs of established theoretical frameworks. For example, the analysis shows that perceived usefulness is a strong predictor of the use of telemedicine technologies, which is consistent with the assumptions of the Technology Acceptance Model (TAM). Perceived ease of use was also significant, with lower technical complexity promoting acceptance. Facilitating conditions such as technical support and training provided by telemedicine centers were identified as crucial for long-term use. These findings confirm the relevance of the theoretical frameworks and offer valuable insights into the factors that influence the acceptance and use of telemedicine technologies. In particular, improvements in perceived quality of care, more efficient organization of daily caregiving tasks, and a reduction in stress and uncertainty were highlighted, outcomes that have repeatedly been described as central benefits of telehealth interventions for informal caregivers [4,6,8,24].

A key finding is that the benefits of telemedicine were not limited to medical monitoring alone. Beyond improved observation of health parameters, family caregivers emphasized greater independence of care recipients and a more structured caregiving routine. Similar multidimensional effects, including psychosocial relief and increased perceived control, have been reported in studies on caregivers of people with dementia, chronic diseases, and palliative care needs [1,2,4,13,25]. These findings suggest that digital technologies may contribute not only to clinical outcomes but also to psychosocial aspects of home care, which are highly relevant for long-term caregiving situations.

The results further show that the integration of telemedicine technologies into everyday care often requires an initial adjustment period. Data protection concerns, technical difficulties, and uncertainty regarding correct use were reported as relevant barriers, particularly at the beginning of use. These barriers closely mirror those identified in international reviews on telemedicine adoption, which emphasize usability, digital literacy, and trust as key determinants of acceptance [14,15,17,26]. However, the reported duration of use in the present study indicates that these challenges can be overcome over time, suggesting a learning process and increasing acceptance once caregivers become familiar with the technologies [14,26].

Another important result concerns communication with healthcare professionals. Family caregivers described communication with physicians and nursing staff as more efficient and targeted when telemedicine technologies were used. Direct access to current health data enabled better preparation for consultations, reduced the need for repeated explanations, and facilitated shared decision-making. Comparable improvements in communication and coordination have been described in studies focusing on oncology, dementia, and palliative care settings [2,12,13,18]. This finding underlines the potential of telemedicine to enhance continuity of care and collaboration across sectors.

### 4.2. Comparison with the Existing Literature

The findings of this study are largely consistent with existing research on telemedicine and digital health interventions in home care settings. Multiple systematic reviews have demonstrated that telehealth can support monitoring, enable timely interventions, and reduce caregiver burden, particularly in dementia and chronic disease care [1,2,5,7,10,25]. The present study confirms these effects from the perspective of family caregivers and adds empirical evidence on perceived everyday benefits and acceptance in real-world caregiving situations. These findings suggest that the effectiveness and perceived usefulness of digital health applications may vary depending on the clinical picture, underscoring the need for a more nuanced assessment of different types of technology. These findings suggest that the effectiveness and perceived usefulness of digital health applications may vary depending on the clinical picture, underscoring the need for a more nuanced assessment of different types of technology.

In line with policy initiatives such as the Digital Care Act and broader national and international digital health strategies, the results support the assumption that digital applications can contribute to more efficient, cross-sectoral care [27,28]. Previous studies have highlighted the potential of telemedicine to address structural challenges in outpatient and home care, including limited access to services and high organizational demands on informal caregivers [6,8,10,26]. The high recommendation rates observed among users in this study further reinforce these findings.

At the same time, the results reflect challenges that are well documented in the literature. Barriers related to interoperability, technical stability, and insufficient training have been repeatedly identified as limiting factors for effective implementation [8,15,16,26,29]. The difficulties reported by some respondents regarding system integration and collaboration between different actors correspond closely with these findings and emphasize the need for organizational and structural solutions beyond the mere provision of technology [30,31].

### 4.3. Role of Structured Support and Telemedicine Centers

A central contribution of the present study is its focus on the need for structured support in the use of telemedicine technologies. Both the quantitative results and the qualitative case study indicate that family caregivers benefit substantially from accessible, continuous assistance. The strong interest in a telemedicine center expressed by respondents reflects the perceived need for a reliable point of contact that combines technical, organizational, and health-related expertise [18,20,21].

The case study illustrates how structured support can influence the successful integration of telemedicine into everyday care. Continuous guidance, rapid problem-solving, and clear explanations increased the caregiver’s confidence and sense of security and enabled sustained use of the technology. The qualitative case study therefore primarily serves an illustrative and explanatory function by highlighting mechanisms rather than offering generalizable conclusions. Similar findings have been reported in studies examining telemedicine centers and coordinated support models, which highlight the importance of human mediation and trust-building alongside technological solutions [20,21,23].

Telemedicine centers could therefore function as central coordination and support units, acting as intermediaries between family caregivers, healthcare professionals, and technical systems. By providing training, individualized advice, and ongoing assistance, such centers may help reduce barriers to adoption, address data protection concerns, and promote long-term use [16,20,30]. From a practical perspective, these findings support the notion that the effectiveness of telemedicine depends not only on technological functionality but also on the availability of supportive infrastructures.

### 4.4. Limitations

Several limitations should be considered when interpreting the results of this study. First, the relatively small sample size limits the generalizability of the findings. While the results provide valuable insights into caregiver experiences, they cannot be assumed to represent all family caregiving contexts.

Second, participants were recruited primarily through caregiving courses and social networks, which may have resulted in selection bias. Respondents may have had a higher interest in digital technologies or greater openness toward telemedicine than the broader population of family caregivers, as also discussed in previous research [17,22].

Third, the study relied on self-reported data, which may be influenced by recall bias or socially desirable responses. In addition, contextual factors such as regional differences, disease severity, and availability of local support services were not systematically examined and may affect transferability.

### 4.5. Implications for Practice and Future Research

Despite these limitations, the study provides important implications for practice and research. The findings suggest that telemedicine technologies can meaningfully support family caregivers when they are user-friendly, tailored to individual care situations, and accompanied by adequate training and support [14,26,32]. Healthcare systems and policymakers should therefore consider not only the provision of digital tools but also the development of supportive infrastructures.

For practice, this includes low-threshold training programs, transparent information on data protection, and easily accessible technical assistance. Telemedicine centers or comparable support structures could play a key role in facilitating access, building trust, and ensuring sustainable use of digital health applications in home care [18,20,21,28]. At the structural level, telemedicine centers require sustainable financing models, clear integration pathways into primary care and specialist services, and standardized approaches to promoting digital literacy in order to enable scalability and long-term implementation.

Future research should include larger and more diverse samples and investigate long-term effects of telemedicine use on caregiver burden, quality of life, and care outcomes. Longitudinal and mixed-methods designs would be particularly valuable to capture changes over time and to better understand how digital technologies can be effectively embedded in everyday caregiving routines [9,19,29,33]. In addition, future studies should supplement self-reported data with objective usage measurements such as application logs or system-generated usage statistics in order to strengthen the validity of the results.

## 5. Conclusions

### 5.1. Summary of Key Findings

The study showed that telemedicine and digital health applications offer significant advantages for the home care of people in need of care. Among the most important findings is that these technologies can improve the quality of care and significantly reduce the burden on family caregivers. The increased independence of those in need of care and more efficient communication with medical professionals are key factors that make everyday care easier.

Integrating the technologies into everyday care was a challenge for many family caregivers, but one that could be overcome over time. Data protection concerns and technical handling were other factors that influenced integration. The use of technology has greatly facilitated and improved communication with doctors and nursing staff, leading to more efficient and precise care.

Cooperation between different digital health solutions and DiGAs is often problematic, particularly due to a lack of uniform interfaces and technical issues. However, many users only use a single app or DiGA, which means that no integration problems arise. The helpful functions and features of the DiGAs used include direct transfer of health data, reminder functions, user-friendliness, motivational games, and safety messages.

The majority of family caregivers would recommend the use of telemedicine technologies, with most users having had positive experiences. The technologies are predominantly rated as good to very good, indicating a high level of satisfaction. The additional comments and remarks make it clear that the technologies are perceived as helpful and facilitating, although they may take some getting used to at first.

### 5.2. Outlook

For future research, it would be useful to expand the study to larger and more diverse samples in order to increase the generalizability of the results. In addition, longitudinal studies should be conducted to examine the long-term effects of telemedicine use on the quality of life of care recipients and their relatives.

With regard to the further development of telemedicine centers, it is recommended that such facilities be established as central points of contact for training and support. These centers could help break down technological barriers and promote the acceptance of telemedicine. By providing comprehensive support and promoting technological literacy, telemedicine centers could make a decisive contribution to improving home care and facilitating the integration of new technologies into everyday life. In order to make the contribution more feasible for healthcare planners, sustainable financing models should be developed to ensure the long-term support and operation of telemedicine centers. This could be achieved through public funds, insurance contributions, or private investment. Stable financing is crucial to providing continuous technical and human resources. In addition, telemedicine centers should be seamlessly integrated into primary care to enable continuous care and coordination between general practitioners and specialists. This requires the development of standardized protocols and communication channels that ensure effective collaboration between different healthcare providers. Finally, standardized training programs should be developed to promote the digital skills of caregivers and family members. These programs should be easily accessible and tailored to the specific needs of users. Regular training courses and workshops can help increase digital literacy and promote the acceptance of telemedicine technologies.

Overall, the findings show that telemedicine should not only be viewed as a technological innovation, but also as an essential tool for improving home care, which should be more widely used in practice. The implications for practice also include the need to remove technological and organizational barriers in order to facilitate access to telemedicine for all stakeholders. This could be achieved by providing comprehensive training and technical support to give family caregivers the necessary confidence and competence in using these technologies.

## Figures and Tables

**Table 1:** Items of the questionnaire.

Main Topic	Example Items	Objective/Measurement Content
Use of telemedicine technologies	“Do you currently use technologies to support home care?”/“Which technologies do you use?”	Recording the frequency of use and typology of systems used
Clinical picture and scope of care	“What is the clinical picture of the person receiving care?”/“How long and how intensively do you provide care?”	Contextualization of application scenarios
Experience and integration	“How easy or difficult was it to integrate DiGAs into everyday life?”/“How long have you been using the technologies?”	Determining acceptance and adaptation difficulties
Impact on care and communication	“Has the quality of care improved?”/“How has communication with doctors changed?”	Assessing perceived effectiveness
Support from telemedicine centers	“Would you find a TMZ helpful?”/“What services would you like to see?”/“What advantages do you see in the connection?”	Exploration of expectations, needs, and potential benefits of a TMZ
Sociodemographic data	Age, gender, level of education, duration of care	Description of the sample

Open questions allowed for qualitative additions (e.g., examples, experiences, suggestions for improvement).

**Table 2:** Digital health applications and software solutions used in family caregiving and reasons for their selection.

Category	DiGAs/Software Solutions Used	Frequency of Mentions	Reasons for Selection
Vital signs & health monitoring	Cardio app, Orbis, mySugr, Apple Health, pedometer, digital VZ measurement	7	Maintain overview, security, control, quick health status
Organization & documentation	Digital calendar, reminder app, Onko app, digital diary, cloud	6	Do not forget anything, relief, better preparation for doctor’s appointments
Therapy, exercise, and training apps	Vivira, memory games app, Casenio	4	Motivation, promote exercise, memory training, fun
Communication & telemedicine	Zoom, Doctolib, video apps	3	Simplified communication, quick coordination with doctors
Emotional/informal support	Conversations, communication without technology	1	Well-being, personal closeness
No use/no information provided	“No response”	1	-
External requirements & framework conditions	Clinic systems, specified tools	3	Clinic standard, availability, acceptance

**Table 3:** Overview of the perceived use of digital technologies in home care, including perceived barriers.

Category	Brief Description	Frequency of Mentions	Typical Statements/Content
Easy integration	Use is perceived as easy, intuitive, and straightforward.	8	“Very simple,” “easy,” “self-explanatory,” “at the touch of a button,” “clear”
Initial difficulties with settling in	Unfamiliar or tedious at first, but becomes routine later on.	7	“Getting used to it,” “difficult at first,” “now part of everyday life,” “stick with it”
Technical and organizational conditions	Acceptance, data protection, external influences.	3	Data protection concerns, not everyone uses everything, coronavirus as a driver of use
Emotional/professional stress	Integration as an additional mental challenge.	1	“It’s hard to get used to,” “you have to love this job”
No difficulties	No challenges perceived.	1	“None,” “It’s OK”
No information provided	No response.	1	“No response”

**Table 4:** Overview of the perceived improvement in home care through the use of digital technology and the effect on communication with medical and nursing staff.

Category	Positive Effects	Specific Examples	Effects on Communication
Health & safety	Better monitoring of health status	Early detection of falls, high blood pressure alerts, blood sugar adjustment, continuous VZ monitoring, rapid response to deterioration	Doctors see values directly, fewer queries, better preparation
Organization & error prevention	Less uncertainty	Reminder app prevents missed appointments, correct medication settings, structured oncology app, digital symptom diary	Fewer errors, everything documented, time savings
Independence & motivation	Greater independence for the person requiring care	Pedometer motivates exercise, memory game boosts motivation, calendar helps with orientation	Nursing staff can track progress
Communication & cooperation	Improved communication with service providers	Video consultations helpful, medical team always up to date	Conversations easier, shorter, data-based, more efficient
Emotional relief & well-being	Reduction in stress and uncertainty	Calm conversation with dementia patients, less stress thanks to orientation and overview	Provides security, facilitates conversations
Limited/no effect	–	“No,” “No answer”	Rejection by nursing staff, skepticism about benefits

**Table 5:** Final assessment of the use of digital technologies.

Category	Number of Mentions	Statements
Helpful/makes everyday life easier	8	“Overall very helpful, makes everything easier.” “Very practical, makes appointments and medication planning easier.” “Good for everyday life, saves a lot of time.” “Very helpful, especially for keeping track of doctor’s appointments.” “Helps me keep track of things, which is reassuring.” “Everything already described.” “No, everything has already been said, the questions are often redundant…” “Nine.”
Motivation & fun	2	“She enjoys it, it motivates her even more.” “Motivates him to go out more, a little help in everyday life.”
Initial difficulties/adjustment period	2	“Works well, just takes some getting used to at first.” “Sometimes annoying, but better than doing everything manually.”
Safety & control	1	“Gives me peace of mind that everything is okay at home.”
No/neutral comments	5	“No answer.” “Everyone has their own opinion.” “No.” “No.” “No.”

**Table 6:** Overview of potential advantages of connecting to a telemedicine center, including desired functions of the telemedicine center.

What Specific Advantages Do You See in Connecting to a Telemedicine Center for Your Daily Care Work? (Question 1)	Number (Question 1)	Are There Any Specific Features or Services You Would Like to See from a Telemedicine Center to Make Your Work as a Caregiver Easier?
Technical support for setting up and using devices and software	6	Technical assistance with electronics and setup Broad technological expertise and experience Faster, more straightforward clicks Simpler calendar function
Quick help with technical problems or using the software	5	Secure notifications in case of problems Automatic updates for appointments/medication Flexible reminder settings
Training courses and workshops on using technologies	4	Exercise instructions directly on your cell phone Game adaptations for memory training Motivation tips for exercise Perhaps consultations, books
Advice on selecting suitable technologies for clinical pictures	3	Advice on purchasing systems Knowledge about diseases Tips on what is useful in telemedicine

**Table 7:** Overview of concerns regarding the telemedicine center and potential benefits.

Category	Advantages	Need for Support	Concerns
Technical support & usability	Reliable setup, smooth and fast use	Hotline, tutorials, step-by-step support	Complexity, technical failures
Advice & guidance	Guidance on suitable technologies	Explanations, videos, individual advice	Fear of making mistakes
Organization & relief	Less stress, better overview	Help with reminders and calendars	Time burden, too many notifications
Health data & monitoring	Better data use, trends, early warnings	Support with data entry and sharing	Too many values, confusion
Communication & coordination	Transparent communication, better physician overview	Data sharing and networking	Dependence on internet
Security & trust	Increased safety	–	Data protection concerns
Motivation & activity	Higher motivation, more activity	Support with exercises and apps	Doubts about motivation effects

**Table 8:** Overview of the expected quality improvement through regular consultations and the requirements for the design of the telemedicine center.

Category	Expected Quality Improvement Through Regular Consultation	Design Requirements for the Telemedicine Center
Communication & exchange	Better exchange, improved error management, less confusion in care	Easily reachable (app, web, phone), chat function, hotline
Monitoring & clinical oversight	Better overview of values, faster identification of trends and problems	Graphics and summaries of values, quick alerts
Documentation & transparency	Better documentation, up-to-date information for physicians	Centralized data storage, easy data sharing with physicians
Learning & quality improvement	Learning effects, supervision, identification of frequent mistakes	Tutorials, short and easy-to-understand instructions
Organization & coordination	Better adherence to appointments	Central contact person, clear and structured interface
Usability & accessibility	Reduced confusion, easier handling	Simple step-by-step use, not too much information, accessible for people with language barriers or low technical affinity
Motivation & engagement	Sustained motivation, increased activity	Motivation tips, small tasks, understandable for older users
Support & responsiveness	Quick help in case of problems	Fast notifications, one-stop support

## Data Availability

The data are contained within the article.
